# Humble Leadership and Team Innovation: The Mediating Role of Team Reflexivity and the Moderating Role of Expertise Diversity in Teams

**DOI:** 10.3389/fpsyg.2022.726708

**Published:** 2022-04-18

**Authors:** Xinghui Lei, Wei Liu, Taoyong Su, Zhiwen Shan

**Affiliations:** ^1^School of Economics and Management, Tongji University, Shanghai, China; ^2^School of Business Administration and Customs Affairs, Shanghai Customs College, Shanghai, China

**Keywords:** team innovation, humble leadership, team reflexivity, team expertise diversity, social information processing theory

## Abstract

The current study proposes a moderated mediation model to explain the relationship between humble leadership and team innovation. Our hypothesis integrates social information processing (SIP) theory with the existing literature on humble leadership. As a result, we theorize that when a humble individual leads a team, the team members are more likely to reconsider strategies, review events with self-awareness, share diverse information, and adapt to new ideas, which in turn promotes innovative team activities. Moreover, consistent with the research that emphasizes the inclusion of team culture in exploring leader–innovation relationships, we investigate the moderating role of a team’s expertise diversity in the above positive, indirect relationship. We test our model by using both archival and survey data collected from 135 teams within 18 medium-to-large internet technology firms in China. The findings largely support our theoretical assertions, suggesting that humble leadership has important implications for team processes and innovation.

## Introduction

In recent years, theories and research into leadership have clearly moved from a strong emphasis on leaders’ special or unusual characteristics toward a focus on the shared growth of leaders and employees. Most recently, humble leadership, a bottom-up and non-traditional leadership approach, has been considered a vital part of emerging leadership theories. Humility has historically been viewed as a philosophical virtue that is important to human excellence (Collins, 2001). Defined as an interpersonal attribute, humility is manifested through accurate self-awareness, appreciation of others’ strengths, and openness to new insights (Nielsen et al., 2010; Owens et al., 2013). Considering the growing number of corporate scandals attributable to the unbridled ego, hubris, and sense of self-importance of organizational executives (Boje et al., 2004), humble leadership has received increasing theoretical elaboration and empirical attention in recent years (e.g., Cameron et al., 2003; Ou et al., 2014). Scholars have revealed the positive effects of humble leadership on followers at the individual and the team level. For example, they have considered employee job satisfaction, work engagement (Owens et al., 2013), relational energy (Wang L. et al., 2018), follower growth (Owens and Hekman, 2012), team effectiveness, and team performance (e.g., Chiu et al., 2016; Rego et al., 2018).

Although previous studies have established a link between humble leadership and employees’ innovation on the individual level (Wang Y. et al., 2018; Zhou and Wu, 2018), they have not considered how humble leadership fosters team innovation. This is an important gap because teams have become the basic building blocks of work in many contemporary organizations. Moreover, general agreement that teams must be innovative to maintain and enhance effectiveness (West and Anderson, 1996; De Dreu, 2002) has implications for exploring factors that contribute to team innovation. To build on, yet differentiate our study from previous research into humble leadership and followers’ individual innovation, we propose a novel model to explore how humble leadership stimulates team innovation.

A considerable number of studies have highlighted team mechanisms that explain the relationships between humble leadership and team outcomes (e.g., collective promotion focus, Owens and Hekman, 2016; team humility, Rego et al., 2017). However, there has been little research that examine the innovation-relevant team processes that humble leadership can facilitate. Social information processing (SIP) theory suggests that followers understand their work environments by processing social information, which in turn shapes their work-related attitudes and behaviors (Salancik and Pfeffer, 1978). Within the SIP framework, leaders are vital sources of information and model how team members should function (Shamir et al., 1993). In this research, we contend that humble leadership results in followers sensing that the team needs continuous improvement and has great potential in the long term. Such perceptions foster an atmosphere of team reflexivity, which is characterized by the pursuit of self-correction and self-transcendence (Johnson et al., 2011; Owens and Hekman, 2012). Team reflexivity is an asset for a team that involves both reflection upon previous strategies and adaptation to prepare for future actions (De Jong and Elfring, 2010). Consequently, exploring the impact of humble leadership on team reflexivity is crucial to create a holistic understanding of how humble leadership influences team effectiveness. In addition, the value of team reflexivity for a team’s innovative performance has been well documented (e.g., West, 2000; Tjosvold et al., 2004; Schippers et al., 2015). Therefore, we seek to uncover the mediating role of team reflexivity between humble leadership and team innovation.

What can ensure that a team with a humble leader engages in a reflection process? Prior research has identified the moderating factors that enable humble leaders to promote group outcomes, such as the power distance of the team, leader member exchange differentiation, and the strength of the team’s psychological capital (Rego et al., 2017; Hu et al., 2018; Carnevale et al., 2019). These previous works have emphasized the moderating effects of subjective contextual factors, devoting less attention to the role of objective team structure, such as a team’s expertise diversity (Kozlowski and Bell, 2012; Hu and Judge, 2017). As a key component in the workplace, team’s expertise diversity governs the extent to which team members’ skills are applied to team tasks (Hansen and Levine, 2009; Hülsheger et al., 2009). According to SIP theory, a proximal working environment provides cues that individuals utilize to construct and interpret work processes (Salancik and Pfeffer, 1978; Jones and Skarlicki, 2005). Hence, team’s expertise diversity invites a wider range of perspectives and legitimizes in-depth processing of task-relevant information (Hansen and Levine, 2009; Hülsheger et al., 2009; Kearney and Gebert, 2009). This may strengthen the impact of humble leadership and promote the behaviors that constitute team reflection (West, 1996, 2002; Van Knippenberg and Schippers, 2007). Therefore, we theorize that team’s expertise diversity, which facilitates more thorough consideration of a team’s functioning, may moderate the effect of humble leadership on team reflexivity.

We further extend this theoretical model by depicting the moderating role of expertise diversity on the indirect effect of humble leadership on team innovation through team reflexivity. Specifically, we hypothesize that the mediated effect of team reflexivity is stronger in the context of a high level of expertise diversity, thus leading to enhanced participation in innovative activities.

The purpose of this paper was to develop a team-level model to explain how and when humble leadership promotes the whole team’s reflexivity and leads to team innovation, making three contributions to the literature. First, we extend the documented positive consequences of humble leadership by focusing on team innovation. This nexus is clearly implied by the rationale behind the effect of humble leaders on team innovation yet has rarely been investigated in empirical humble-leadership research. Second, based on SIP theory, our study contributes to the growing humble-leadership literature by detailing how humble leadership impacts team innovation *via* team reflexivity. Identifying the mediating role of team reflexivity in the humble leadership–team innovation link is particularly important because doing so creates a comprehensive picture of the function and influences processes of humble leadership. Third, by proposing the boundary conditions of humble leadership, this study furthers our understanding of how humble leadership fosters team reflexivity (Son, 2020). Exploring team’s expertise diversity as a critical contingency for humble leadership’s impact on team reflexivity helps illuminate why some humble leaders promote team reflexivity while others do not, providing insight into the limits of humble leadership.

**FIGURE 1:**
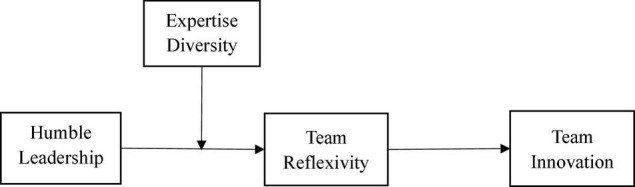
Hypothesized direct and indirect relationship in this study.

Taken together, these investigations (as summarized in Figure 1) reveal the theoretical potential of humble leadership, which enables leaders to influence their followers to form reflective and innovative collectives and clarify the boundary conditions for the impact of humble leadership. In doing so, we provide practical suggestions for organizations to encourage reflection processes and high-level innovation at the team level. In view of the current demand for more fairness, free management and leadership inspired by the ideas of humble leadership theory may very well be what organizations need now.

## Theoretical Background and Hypotheses

### Humble Leadership

Humility, a virtue in psychology, comes from the Latin words “humus” and “humi,” meaning “soil” and “on the ground,” respectively, with the focus on viewing oneself and others from an inferior standpoint (Owens and Hekman, 2012). People with humility are modest and inclined to learn from their surroundings (Morris et al., 2005). Contemporary scholars treat humility as a moral skill that can be developed or a moral muscle that can gradually change through experience or training (Bloomfield, 2000; Stichter, 2007, 2011). As used by Cameron et al. (2003), humility is a key organizational virtue, reflected in the pursuit of consistent development and the appreciation of employees (Tangney, 2000; Owens and Hekman, 2016). Moreover, in practice, scandals caused by the unbridled ego and hubris of entrepreneurs (e.g., Boje et al., 2004) have demonstrated the significance of humble leadership for a firm’s longevity (Weick, 2001, p. 93). An accurate definition of humble leadership was proposed by Owens et al. (2013), leading to many interpretations that exemplify a wide range of behaviors. Owens and Hekman (2016) distinguished three characteristics that are generally quoted as the essential elements of a humble leader: (1) admitting their shortcomings, (2) appreciating the advantages or contributions of others, and (3) being open to feedback. Leaders’ humility is also theoretically centered on the concept of self-transcendence, which refers to seeing and pursuing value beyond the self (Morris et al., 2005; Ou et al., 2014).

Based on these psychological and philosophical roots, scholars have made substantial strides toward empirical rigor regarding positive follower outcomes, such as in-role performance (e.g., Owens et al., 2013), helping behavior (Carnevale et al., 2019), and focus on collective promotion (Owens and Hekman, 2016). According to SIP theory, a leader’s display of humility substantially influences followers’ energy and orientation by providing social cues and serving as an archetype (Owens and Hekman, 2016; Wang L. et al., 2018). In contrast to transformational leadership, ethical leadership, and servant leadership, humble leaders focus on improving themselves and developing followers, with greater allegiance to long-term performance goals (Mao et al., 2019). With respect to the inclusive and promotion-focused information provided by humble leaders, our research aimed to examine the implications of humble leadership for team innovation by enrolling a mediating variable—team reflexivity. By admitting mistakes, appreciating others’ strengths, and being open to advise, humble leaders shape their followers’ perception that reflective discussions about the team’s goals and weaknesses are legitimate, laying important groundwork for the process of team reflexivity.

### Team Reflexivity

Team reflexivity is defined as a transition phase process in which group members reflect upon and communicate about the group’s objectives and strategies and adapt them to anticipated circumstances (West, 1996). Conceptual and empirical evidence (LePine et al., 2008) suggests that reflexive behaviors, such as “questioning, learning at a meta-level, reviewing past events, and coming to terms over time with a new awareness” (West, 2000), can help teams to derive good from bad situations and improve task effectiveness (Carter and West, 1998). As a team asset, team reflexivity focuses on both reflection upon previous accomplishments and adaption to prepare for future actions (Schippers et al., 2003). Research regarding the value of team reflexivity has treated team reflexivity as “a form of strategy formulation and planning” that functions to improve subsequent team performance (Bass, 2000; Vashdi et al., 2007). Likewise, a large body of work has found that team reflexivity is positively associated with other team outcomes, such as team productivity, team effectiveness (e.g., De Dreu, 2007), and employees’ affective wellbeing (Carter and West, 1998).

While team reflexivity has been primarily demonstrated to be a useful tool for promoting positive team outcomes, a growing number of studies have suggested that exploring the determinants of team reflexivity is also important (De Jong and Elfring, 2010; Schippers et al., 2015; Lyubovnikova et al., 2017). The antecedents of team reflexivity have been proposed to include clear team goals (Dayan and Basarir, 2010), moderate time pressure (Schippers et al., 2014), task interdependence, and psychological safety (Edmondson and Lei, 2014). These determinants seem to imply that team reflexivity is induced by an integrative climate and environmental opportunities, which are mostly influenced by team leaders. Thus, the role of leadership in facilitating team reflexivity appears to be a promising avenue for investigation. Indeed, participative leadership (Somech, 2006), facilitative leadership (Hirst et al., 2004), and transformational leadership (Schippers et al., 2008) have been found to motivate the team reflection process. However, despite common theoretical underpinnings on these leadership approaches, previous research has not considered that humble leadership may play a unique role in engendering this process. Compared with other traditional leadership styles, humble leaders provide social cues (self-correction and self-transcendence) that are more consistent with team reflexivity.

### Humble Leadership and Team Reflexivity

Social information processing theory indicates that team managers’ psychological and behavioral cues are influential in shaping perceptions about norms of acceptable behaviors during work processes (Shamir et al., 1993). Researchers have empirically demonstrated that team leaders signal followers to understand their working environment and encourage certain behaviors in the workplace (e.g., Yaffe and Kark, 2011). Drawing from SIP theory (Salancik and Pfeffer, 1978), we note that team members recognize the social cues provided by the leader that displays humility and then develop a perception of legitimate development and growth (Owens and Hekman, 2012, 2016). Such a perception is conducive to cultivating the reflective atmosphere of the whole team (Salancik and Pfeffer, 1978). Specifically, as humble leaders acknowledge the team’s imperfection in previous tasks and put accomplishments in perspective (Landrum, 2011), they encourage subordinates to accept their limitations and pursue improvement, legitimizing team members’ self-acceptance and self-transcendence. Thus, we can link the relationship between humble leadership and team reflexivity with SIP theory.

The theoretical foundation of humble leadership implies that humble leaders exemplify an awareness of self-limitations and show openness to feedback (Vera and Rodriguez-Lopez, 2004; Ou et al., 2014). Their legitimization of failures and appreciation of advice connote the belief that a continual evaluation and revision of team validity will enable team members to “identify, clarify, and pursue the way to success” (Rego et al., 2019). With these cues, humble leaders encourage employees to face negative team performance objectively and to ask questions, evaluate limits, and seek progress, rather than to hide problems or to insist on the utilization of conventional processes (West, 2000). Furthermore, humble leaders recognize and validate the unique strengths of their followers (Morris et al., 2005), which motivates team members to accept and compliment partners’ contributions and further legitimizes sharing and coping within the workplace (Ou et al., 2018; Wang L. et al., 2018). Such constructive interaction strategies allow for comprehensive and systematic assessments of performance problems (West, 2000), enabling team members to share knowledge and develop more effective ways to overcome challenges. In addition, by stimulating their followers’ sense of self-efficacy and self-worth (Johnson et al., 2015), humble leaders lead followers to generate adaptive attitudes toward frustrations and an optimistic view of novelty, which are also key factors in reinforcing team reflexivity.

The above arguments propose that humble leaders allow team members to reflect on the past and prepare for the future. In contrast to leaders who are narcissistic or claim excessive credit for the success of the group, a leader displaying humility encourages followers to spend time in reflective activities. This includes reflecting on the current level of task effectiveness and considering how the content and suitability of their objectives and processes align with the team’s true values and intentions. Based on these arguments, we propose the following hypothesis:

***H1: Humble leadership will be positively related to team reflexivity.***

### The Mediating Role of Team Reflexivity

We further assess the implications of team members’ reflections on the past by examining team innovation as a distal outcome of humble leadership *via* team reflexivity. The extant humility literature has suggested that teams with humble leaders are characterized by open-mindedness concerning new things (Wang L. et al., 2018). In addition, humble leadership is demonstrated to enhance employee innovation because acceptance of criticism creates an inclusive organizational atmosphere (Zhou and Wu, 2018) and self-transcendence stimulates engagement in challenging development activities (Wang Y. et al., 2018). As a result, humble leadership may provide opportunities for innovation at the team level by legitimizing errors and creating novel ideas for development.

When followers are influenced by a humble leader who is engaged in a team reflection process, they offer a source of competitive advantages for the sustainable development of the team or organization (Schippers et al., 2015). As an important factor to indicate team sustainability, team innovation, which addresses the challenges and shocks that teams encounter (Hirst et al., 2009), can be prompted by the processes of team reflexivity (Schippers et al., 2015; Wang et al., 2020). Indeed, Carter and West (1998) emphasize that task reflexivity is positively related to participation in decision-making, flexible strategies and objectives, and positive relational energy among followers, thus promoting a team’s effectiveness and innovation.

Specifically, team reflexivity involves reflecting upon the group’s past performance, objectives, and methods and considering new ways to adapt to anticipated environments (West, 1996). Teams with high levels of reflexivity are more innovative because they (a) reflect on previous work processes so that team members abandon less-promising ideas in favor of better options (De Dreu, 2002); (b) engage in deep communication about the experience gained from the initial team performance, which is conducive to problem-solving and the development of new techniques; and (c) improve their ability to effectively utilize prior feedback and continually promote the incubation of new ideas (Paulus and Yang, 2000). In short, teams that engage in such deep processes (team reflexivity) are more likely to develop learning behaviors that lead to the successful accomplishment of goals and additional team innovation.

In summary, the preceding discussion suggests that humble leaders’ behaviors improve team reflexivity, which, in turn, contributes to team innovation. We accordingly hypothesize the following:

***H2: Humble leadership will have a positive, indirect effect on team innovation via team reflexivity.***

### The Moderating Role of a Team’s Expertise Diversity

The interactive effects of a team’s functional diversity and leadership have been investigated by many studies (e.g., Dahlin et al., 2005; Kearney and Gebert, 2009), but the focus has been on how leaders manage diversity in teams and the effect on performance. In this regard, there is a need to explore the moderating role of a team’s expertise diversity in the relationship between humble leadership and team reflexivity.

Social information processing theory suggests that social cues from the environment affect team process directly and indirectly *via* interactive activities (Salancik and Pfeffer, 1978; Chen et al., 2013). Individuals in a functionally diverse team can experience a wide range of perspectives and promote dissent and valuable debates (Williams and O’Reilly, 1998), pursuing the acquisition of disparate knowledge and information (De Dreu, 2007; Cabrales et al., 2008). Drawing on SIP theory, we posit that expertise diversity provides potential opportunities for group-thinking and knowledge-sharing, which enhance a humble leader’s promotion of continual team reflexivity. Expertise diversity, which refers to the heterogeneous functional backgrounds of team members (Jackson, 1992), determines whether the followers’ functional backgrounds constitute an enlargement of the team’s pool of knowledge, information, and perspectives (Jackson, 1992; Simons et al., 1999; Jackson and Joshi, 2004).

As noted earlier, when humble leaders motivate team members to reflectively consider the past, expertise diversity facilitates the practical application of team reflection. This is because team members who are exposed to various experiences and perspectives (De Dreu, 2002) have more opportunities to deeply reanalyze work and questions. Expertise diversity represents the potential for frequent communication, constructive conflict, and groupthink; in turn, these factors enhance the effect of humble leadership (Schippers et al., 2003). When the level of expertise diversity is high, team members are provided with insights from different perspectives and engage in a more effective consideration of team functioning, thus improving the feasibility of team reflexivity (Van Knippenberg and Schippers, 2007). The higher the level of expertise diversity in a team, the more knowledge resources are available to facilitate the cross-fertilization of ideas (Perry-Smith, 2006; Hülsheger et al., 2009). Teams with high levels of expertise diversity have access to a more varied set of frameworks than those with low levels of expertise diversity, allowing them to thoroughly analyze more information. Overall, expertise diversity presents an open and broad context for team followers that can refresh their cognitive resources and strengthen the modeling effect of humble leadership.

Consequently, when employees have access to diverse knowledge, they are highly capable of emulating their humble leaders by reflecting on the past. We thus suggest that humble leadership tends to be more effective in legitimizing the team reflection process when the level of team’s expertise diversity is high. Specifically, we hypothesize the following:

***H3: Team’s expertise diversity moderates the humble leadership–team reflexivity relationship, so that this positive relationship is stronger when the level of expertise diversity is high.***

The prior arguments represent an integrated framework in which team reflexivity mediates the relationship between humble leadership and team innovation and the effect of humble leadership on team reflexivity depends on the level of team’s expertise diversity. This hypothesized pattern implies moderated mediation, whereby an indirect effect varies as a function of a third variable (Edwards and Lambert, 2007). Based on this, we further hypothesize that team’s expertise diversity also moderates the mediated relationship between humble leadership and team innovation *via* team reflexivity; that is, this impact implicates a moderated mediating effect. Therefore, we hypothesize the following:

***H4: Team’s expertise diversity moderates the mediating effect of team reflexivity on the relationship between humble leadership and team innovation, so that the mediating role of team reflexivity will be stronger for teams with higher levels of expertise diversity.***

## Materials and Methods

### Sample and Procedure

The initial sample consisted of 756 employees who were working in 160 teams within 18 information and technology companies in the south of China. The functions of these teams ranged from hardware testing, program writing, and system analysis to improve the quality of existing products and designing new software for customers. We selected teams in which team members needed to work interdependently and interact frequently to successfully accomplish team tasks. Following Schippers et al. (2007), we did not consider teams with very routine jobs, as team reflexivity and innovation activities were unlikely to be significantly relevant for these teams.

Data were collected through a web survey since the involved teams are located in different cities in southern China. Three authors contacted chief executive officers (CEOs) through their personal networks, and the CEOs encouraged employees to participate in our study. Human resource (HR) departments were accessed by telephone, and we chose teams based on our selection criterion that the team’s work should be interdependent and non-routine. The questionnaires were sent to team members and supervisors by the HR department with a unique firm code provided by the authors and a team code provided by the HR department and researchers. A cover letter explained the purpose of our study and guaranteed the respondents’ confidentiality. All the team members sent the completed questionnaires directly to the researchers. To reduce the risk of common method variance (Podsakoff, 2003), we collected data on the different variables from different sources: the data on demographic variables and expertise backgrounds were provided by the HR departments, the team members reported on humble leadership and team reflexivity, and the team leaders evaluated team innovation. Except for the survey variables, other variables in our model were assessed through the objective measurements of the whole team, which were aggregated from all team members. Hence, once a team participated in the study, we included objective data regarding both the employees completing the questionnaire and those not completing the survey, in harmony with our purpose to attain objective team-level data.

Our overall response rate was 84% and we obtained a sample of 142 teams with leader–team member dyadic data. To better represent the response of the whole team (Langfred, 2004; Cheung et al., 2016) and match questionnaire data with archival data on the team level, we restricted the sample to include only teams with a participation rate over 50% (i.e., more than half of the team members provided complete responses to the survey) and teams where at least three team members responded to the survey. Applying these selection criteria resulted in a final sample of 135 teams, composed of 135 leaders and 669 team members. The size of the teams ranged from 3 to 13 employees (median = 7, standard deviation [SD] = 3.02). Specifically, there were 67 teams with 3–7 members, 35 teams with 7–10 members, and 33 teams with 10 or more members. Additionally, 68% of the respondents were men, and the team members’ average age was 40.25 years old ([SD] = 8.74). On average, the respondents had worked in their current team for 2.85 years ([SD] = 1.27).

### Measurements

We translated all the surveys from English to Chinese following Brislin (1986) approach. Unless otherwise stated, we assessed all the questionnaire items using a five-point scale from 1, “completely disagree” to 5, “completely agree.”

#### Humble Leadership

Humble leadership was measured using a nine-item scale that reflected the three proposed dimensions of humility (Owens et al., 2013). Since highly humble people tend to underrate their own humility and those low in humility tend to overrate their own humility (Tangney, 2000; Ou et al., 2018), we followed previous humble-leadership literature and used other report measures of humble leadership (e.g., Owens et al., 2013; Owens and Hekman, 2016). Sample items included “This leader admits it when he/she does not know how to do something,” “This leader shows a willingness to learn from others,” and “This leader often compliments others on their strengths.” The alpha reliability (α) for this scale was 0.83.

#### Team Reflexivity

Team reflexivity was measured with six items adapted from Carter and West (1998), which were later validated through the work of Schippers et al. (2003). These six items were measured the extent to which team members collectively reflected on their team’s objectives, strategies, and processes. Specific items included “We regularly discuss whether the team is working effectively,” “The methods used by the team to get the job done are often discussed,” “We talk about different ways in which we can reach our objectives,” and “The team often reviews its objectives” (α = 0.81).

#### Expertise Diversity

Information for this measure was provided by each company’s HR department; therefore, expertise diversity was based on the functional composition of each team. In line with Harrison and Klein (2007), we measured this variable *via* Blau (1977) formula, 1 − *p*_*i*_^2^, where *p* is the proportion of a group in the *i*th category within a team. The index ranges from 0, indicating no diversity, to a theoretical maximum of 1; higher index scores indicate greater expertise diversity among team members. Following Bunderson and Sutcliffe (2002), the team members were categorized into nine broad disciplinary areas, such as sales, marketing, manufacturing, or research and development.

#### Team Innovation

To avoid potential common source bias, the supervisors of participating teams were asked to rate the innovation of their teams on a scale from one to five. Team innovation was measured by four innovation criteria based on previous research (Anderson and West, 1998). Sample items were (1) “Team members often implement new ideas to improve the quality of our products and services” and (2) “This team gives little consideration to new and alternative methods and procedures for doing their work (reverse coded)” (α = 0.83).

#### Control Variables

We controlled for team size and the diversity of three of the team members’ main demographic characteristics (gender, age, and job tenure), as these factors have been found to be related to team reflexivity and innovation (Schippers et al., 2003; Hirst et al., 2004; LePine et al., 2008). Team size was measured as the number of members within a team, which has been shown to influence team processes and functioning (Hackman and Vidmar, 1970; Cummings et al., 1974). Gender diversity was assessed by Blau’s index, in which men and women comprise heterogeneous categories. Tenure diversity was calculated as the coefficient of variation (SD divided by the mean) of the number of years team members had spent in the job. Similarly, age diversity was calculated as the coefficient of variation of team members’ ages.

### Preliminary Analysis

#### Data Aggregation

To ensure that data aggregation was appropriate, we calculated within-group inter-rater agreement (r_*wg*_; James et al., 1983) and intraclass correlation coefficients (ICCs; Bliese, 2000) for the follower-rated team constructs, namely, humble leadership and team reflexivity. The r_*wg*_ scores averaged 0.96 for humble leadership and 0.98 for team reflexivity, suggesting that aggregating to the team level was justified (LeBreton and Senter, 2008, p. 836). The ICC1 scores for humble leadership and team reflexivity were 0.24 and 0.35, respectively. The ICC2 values for humble leadership and team reflexivity were 0.61 and 0.72, respectively. The ICC values indicated that a considerable amount of the variation in ratings was due to team membership (Bliese, 2000). Overall, these results justified our aggregation of the responses at the team level.

#### Assessing Discriminant Validity

Prior to hypothesis testing, we first conducted a team-level confirmatory factor analysis (CFA) by using aggregated scores of three key scale constructs: humble leadership, team reflexivity, and team innovation. The results of the CFA suggested that the expected three-factor model fits our data reasonably well [χ^2^(62) = 101.497, *p* < 0.001; root-mean-square error of approximation (RMSEA) = 0.07; standardized root mean residual (SRMR) = 0.02; comparative fit index (CFI) = 0.97]. The fit was superior to models in which (a) team reflexivity and team innovation were combined into one factor [χ^2^(65) = 511.6, *p* < 0.001; RMSEA = 0.27; SRMR = 0.09; CFI = 0.64] and (b) humble leadership and team reflexivity were combined into one factor [χ^2^(64) = 359.904, *p* < 0.001; RMSEA = 0.19; SRMR = 0.06; CFI = 0.76]. Taken together, these results favored the three-factor model, thus supporting discriminant validity among the measures.

## Results

When testing our hypotheses, we needed to consider 18 companies’ attributes to control organization-level effects. Therefore, we adopted random coefficient modeling (also termed hierarchical linear modeling; Raudenbush and Bryk, 2002), using the statistical software R. This helped us to control the effects of unmeasured organizational-level characteristics because random coefficient modeling accounts for the non-independence of nested data. It also partitions the total variance of the outcome variable into the within-firm level (in our case, team level) and between-firm level. To evaluate the goodness-of-fit of the models, we calculated pseudo R-squares (∼*R*^2^) based on Snijders and Bosker (1999), which represent the proportional reduction of errors at all levels after adding predictors to the model. Furthermore, we conducted a supplemental analysis to test the moderated indirect effect of humble leadership on team innovation *via* team reflexivity. Since appropriate methods for bootstrap analysis with multi-level data have yet to be developed, we drew on Hayes and Preacher’s work and formula (Hayes et al., 2011) to construct confidence intervals (CIs) for indirect effects at different levels of team’s expertise diversity.

The means, SDs, and correlations for all the team variables are shown in Table 1. As Table 1 reveals, humble leadership is positively correlated with both team reflexivity (*r* = 0.16, *p* < 0.10) and team innovation (*r* = 0.25, *p* < 0.01). Team reflexivity was also significantly correlated with team innovation (*r* = 0.40, *p* < 0.01).

**TABLE 1:** Descriptive statistics and bivariate correlations.

Variables	Mean	*SD*	1	2	3	4	5	6	7
1. Team size	7.15	3.02							
2. Gender diversity	0.36	0.14	0.20*						
3. Age diversity	0.19	0.05	0.17*	−0.08					
4. Tenure diversity	0.56	0.17	0.09	0.20*	−0.06				
5. Humble leadership	3.71	0.55	0.15	−0.07	0.14	0.06			
6. Expertise diversity	0.55	0.19	0.40**	0.10	−0.05	0.06	0.12		
7. Team reflexivity	3.80	0.43	0.00	0.12	−0.05	0.09	0.16^†^	−0.06	
8. Team innovation	3.96	0.63	0.29**	0.08	0.03	0.03	0.25**	0.07	0.40**

N = 135 teams. Team size is the number of individual members in a team. Team gender diversity = 1 − p_i_ (i = 0 for men, and i = 1 for women). Team age diversity, SD/mean. Team tenure diversity, SD/mean. Expertise diversity = 1 − p_i_ (i represents the category of expertise). ^†^p < 0.10, *p < 0.05,**p < 0.01.

Table 2 presents the results of the models used for hypothesis testing. As indicated in Model 2, after including the controls, humble leadership was positively related to team reflexivity (*b* = 0.15, *p* < 0.05); thus, Hypothesis 1 was supported.

**TABLE 2:** Summary of path-analysis results of hypotheses 1 and 2.

Variables	Team reflexivity			Team innovation				
	Model 1	Model 2	Model 3	Model 4	Model 5	Model 6	Model 7	Model 8
Intercept	3.69*** (0.20)	3.19*** (0.31)	3.12*** (0.30)	3.50*** (0.33)	2.54*** (0.44)	0.87 (0.57)	2.63*** (0.47)	0.98 (0.60)
Team size	−0.00 (0.01)	−0.01 (0.01)	0.00 (0.01)	0.06** (0.02)	0.06** (0.02)	0.06 (0.02)	0.06** (0.02)	0.06** (0.02)
Gender diversity	0.32 (0.27)	0.38 (0.28)	0.37 (0.25)	0.13 (0.37)	0.24 (0.35)	−0.03 (0.33)	0.29 (0.35)	−0.12 (0.32)
Age diversity	−0.37 (0.72)	−0.63 (0.77)	−0.82 (0.68)	−0.02 (1.11)	−0.21 (1.08)	−0.21 (0.89)	−0.69 (1.05)	−0.07 (0.87)
Tenure diversity	0.17 (0.22)	0.12 (0.22)	0.11 (0.21)	−0.04 (0.37)	−0.12 (0.37)	−0.16 (0.35)	−0.20 (0.36)	−0.22 (0.36)
Humble leadership		0.15* (0.07)	0.18** (0.06)		0.28** (0.10)	0.19* (0.08)	0.27** (0.10)	0.18* (0.09)
Expertise diversity			−0.10 (0.23)				−0.00 (0.31)	0.06 (0.28)
Team reflexivity						0.54*** (0.12)		0.52*** (0.12)
HL*ED			1.12** (0.35)				1.05 (0.66)	0.41 (0.68)
∼R^2^	0.02	0.05	0.14	0.05	0.09	0.18	0.11	0.20

Coefficient estimates are based on 135 teams in 18 organizations. Table entries represent unstandardized coefficients with standard errors in parentheses. HL, humble leadership; ED, expertise diversity. ^†^p < 0.10, *p < 0.05, **p < 0.01, ***p < 0.001.

We followed the procedures established by Baron and Kenny (1986) to test Hypothesis 2 regarding the mediating role of team reflexivity in the humble leadership–team innovation relationship. First, humble leadership as evaluated by followers was positively related to team innovation (*b* = 0.28, *p* < 0.01; Model 5). Next, by proving Hypothesis 1, we verified the positive effect of humble leadership on team reflexivity in Model 2. Finally, in Model 6, in which team reflexivity was added, the effect of humble leadership on team innovation became less significant (*b* = 0.19, *p* < 0.05). To further shed light on the indirect effects, we conducted a bias-corrected bootstrap analysis (5,000 samples) with the PROCESS macros developed by Hayes (2013). We found that the indirect effect of humble leadership on team innovation *via* team reflexivity was 0.08, with a 95% CI [0.006, 0.173]. The results revealed that team reflexivity was a partial mediator, supporting Hypothesis 2.

**FIGURE 2:**
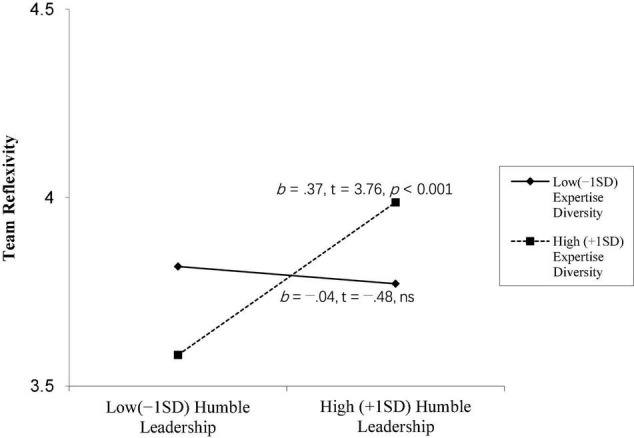
Moderating effect of team expertise diversity.

As shown in Model 3, the interactive effect of humble leadership and team’s expertise diversity on team reflexivity was significantly positive (*b* = 1.12, *p* < 0.01). We plotted the relationships between humble leadership and team reflexivity at high and low levels of team’s expertise diversity (1 SD above and below the mean). In Figure 2, the simple slope tests indicate that the positive effect of humble leadership on team reflexivity is stronger for teams with higher levels of expertise diversity (*b* = 0.37, *p* < 0.001) rather than with lower levels (*b* = −0.04, ns). This significant interaction effect supported Hypothesis 3.

To address Hypothesis 4, which referred to the integrative moderated mediation model, we conducted supplemental analyses by calculating the 95% CI for the indirect effects conditioned at low (−1 SD) and high (+ 1 SD) team’s expertise diversity. The results show that the indirect effect of humble leadership on team innovation *via* team reflexivity was positive when the level of team’s expertise diversity was high (95% CI = [0.088, 0.382]) but not when the level of team’s expertise diversity was low (95% CI = [−0.192, 0.064]). Therefore, Hypothesis 4 was supported.

## Discussion

As humble leadership is considered a critical factor that affects followers’ behavior, existing literature has focused on how employees’ innovation and team performance can be promoted by humble leadership. Our study explored an important mechanism by investigating how and when humble leadership can promote team innovation. By integrating SIP theory with humble leadership research, we predicted that humble leadership plays a significant role in enhancing team innovation by engendering team reflexivity. The predicted mediation model was supported. In response to the recent calls for developing a deeper understanding of humble leadership (Wang L. et al., 2018), this article empirically viewed expertise diversity as an important boundary condition for the effectiveness of humble leadership. It was shown that the effect of humble leadership on team reflexivity tends to be stronger in a team with a high level of expertise diversity. These results highlight the direct and indirect relationships between humble team leadership, team reflexivity, and team innovation at different levels of team’s expertise diversity.

### Theoretical Implications

This article makes several important contributions to the current literature. First, our study offers an extension to the humble-leadership literature by exploring the positive relationship between humble leadership and team innovation. Conventional research has suggested that humble leadership is an emerging organizational concept that has been found to foster positive outcomes within teams (e.g., Owens and Hekman, 2016; Rego et al., 2017; Hu et al., 2018). However, existing research has not included how the behaviors and incentives of humble leaders influence team innovation. To enable a better understanding of the unique role of humble leadership in team innovation, we propose that unlike leaders who employ other leadership styles, humble leaders display certain characteristics. They show endurance in the face of failures, view limitations as opportunities to learn, and emphasize the importance of learning from mistakes, which may create a supportive environment for innovation. Our process model for how leaders stimulate team innovation may help provide insight into how leaders greatly affect organizational core competitiveness.

Second, by underscoring team reflexivity, we provide an alternative to team-mediation mechanisms that are used to explain how humble leadership influences team outcomes. Current studies have posited several team mechanisms, such as orientation, values, and action (Owens and Hekman, 2016; Rego et al., 2017; Chen et al., 2021). However, most have focused on team orientation, limiting our understanding of the actions humble leadership can influence within the team context. Through utilizing SIP theory, we found that subordinates receive and process information from humble leaders, which shapes their reflective attitudes and behaviors. The mediation results are consistent with Schippers et al. (2007, p. 1) who stated, “Leadership is a very relevant factor on determining team reflexivity process.” Schippers et al. (2015, p. 6) further suggested, “Highly reflexive teams will develop more ideas for new and improved ways of doing things or new products and services.” This study has broadened our understanding of how humble leaders can shape team reflection processes and subsequent innovation.

Third, this study represents one of the few empirical tests of the boundary conditions of the effect of humble leadership on team innovation, answering the call of many studies (Owens and Hekman, 2012). This research incorporates team’s expertise diversity and team reflexivity into a single framework, leading to a better understanding of the benefits of humble leadership. Support for our moderation model provides a novel contribution, revealing that when humble leadership is combined with a team’s functional heterogeneity, its positive effect is augmented. This theoretical effort implies that a team’s objective composition establishes the foundation for humble leaders to foster the reflection process of followers. This is consistent with the underlying thesis that teams with an appropriate leadership style and functionally heterogeneous composition have a greater ability to improve effectiveness (Somech, 2006). In addition, this study extends humble leadership research by indicating that team’s expertise diversity helps humble leadership to yield the favorable outcome of team innovation.

### Practical Implications

Companies value creative teams to help in achieving sustainable development in a dynamic environment (Shalley and Gilson, 2004). The explicit mechanism of humble leadership reveals that to promote team innovation team leaders should seek to create a climate facilitating self-transcendence and encouraging reflection on the past. In other words, encouraging leaders to exhibit humble behaviors may help firms to manage the innovation-enhancing processes mentioned in our study. Thus, corporate executives need to conduct work to enhance the level of humble leadership. Practices, such as selecting humble individuals as managerial candidates and training managers in humble behaviors, can help firms to generate and develop more humble leaders.

Second, by demonstrating that team reflexivity acts as a vital cognitive mechanism in team innovation, this study pinpoints a proximal target antecedent of team innovation that humble leadership can promote. However, teams in organizations tend to behave in habitual ways, and Schippers et al. (2015) proposed that it may be energy- and time-consuming for high-performing teams to implement reflexivity. Our results solve the problem by suggesting that reflective activities can become more customary, part of a team’s daily functioning, rather than being intentionally trained. In this respect, leaders should enact humble models to encourage team members to engage in spontaneous reflexivity within the team.

Finally, we show that the positive effects of humble leadership on team reflexivity and team innovation are influenced by the level of team’s expertise diversity. We encourage organizations that want to reap the benefits of humble leadership to consider ways of creating appropriate conditions that can help to enhance the effectiveness of humble leadership. One route is to establish functionally heterogeneous teams, providing team members with a wide range of knowledge and a foundation to enact team behaviors fostered by humble leaders. Otherwise, humble leadership may generate fewer positive effects. Therefore, we encourage organizations to create genuinely challenging goals for teams and executives to assign functionally heterogeneous members to complete complex tasks.

### Limitations and Future Research

Despite our contributions, our research has potential limitations that offer directions for future research. First, one limitation lies in the cross-sectional data used in our study, as this design does not allow the directionality of the results to be tested. Although alternative arguments based on reverse causality assumptions are less likely to be valid from a theoretical standpoint (e.g., it is not clear how team reflexivity could affect humble leadership), we cannot conclusively eliminate them. Therefore, to confirm the causal direction of the relationships, future studies should consider longitudinal research methods, such as conducting three-phase data collection or a field experiment.

Second, the use of a sample from only one country may unfavorably influence the generalizability of our findings and raise questions about the validity of our conclusions. In China, classical culture (Confucian and Taoist) emphasizes the doctrine of the mean and the virtue of humility, which leads people to be praised highly for being humble. Leaders in such a context are more likely to adopt humble behaviors, and followers tend to be less extreme and to continuously reflect on themselves. Future research that could validate this model in different cultural contexts (e.g., Western contexts) would be very valuable.

The third limitation of this study is that while we have evidence to identify the specific mechanism involved, team reflexivity, we have not examined the dynamics of this process. In different situations, distinct pressures and goals may influence teams’ focuses and behaviors during the reflection process (Schippers et al., 2013). For example, teams that experience disappointing performance will focus on learning from previous mistakes and changing their course of action to improve subsequent performance; teams with positive performance feedback tend to communicate with external peers to find their limitations, reflect upon the past, and attain sustainable success. We recommend future research to extend our findings within specific contexts and discuss possible contingencies of reflexivity in relation to innovation. We also encourage future studies to identify the effects of team reflexivity on other group outcomes, such as team efficiency and team growth.

Fourth, to compare innovation across participating teams engaging in different tasks, we employed subjective supervisory ratings to measure innovative achievements. Although such ratings facilitate cross-team comparison, as with any survey-based method, they are subject to human assessment errors. This inclination may translate to giving accurate assessments of team innovation, that is, encouraging tests of objective differences in team innovation in the future.

Last, the negative side of humble leadership has been completely neglected in this study. Although humble leadership has been found to generate insightful implications within organizations, leaders displaying humility also face the risk of being viewed as weak or powerless (Petrenko et al., 2019). For instance, humble female leaders may be given poor leader-effectiveness ratings because of gender bias (Lanaj and Hollenbeck, 2015). Future research might consider the downsides and boundary conditions of humble leadership to yield a more comprehensive understanding of humble leadership.

## Conclusion

The bottom-up leader approach, humble leadership, has received increasing attention in recent research and practice. Our article illustrates that humble leadership enhances team innovation by fostering reflection among followers. This research also shows that team’s expertise diversity moderates the relationship between humble leadership and team reflexivity. In addition, the indirect effect of humble leadership on team innovation (*via* team reflexivity) was stronger when the level of team’s expertise diversity was high. We hope these efforts encourage further research to understand the impact and contingencies of humble leadership.

## Data Availability Statement

The original contributions presented in the study are included in the article/Supplementary Material, further inquiries can be directed to the corresponding author/s.

## Author Contributions

XL and WL: conceptualization. TS: methodology. ZS: formal analysis, investigation, writing—review, and editing. WL: writing—original draft preparation. XL: supervision. All authors contributed to the study’s conception and design.

## Conflict of Interest

The authors declare that the research was conducted in the absence of any commercial or financial relationships that could be construed as a potential conflict of interest.

## Publisher’s Note

All claims expressed in this article are solely those of the authors and do not necessarily represent those of their affiliated organizations, or those of the publisher, the editors and the reviewers. Any product that may be evaluated in this article, or claim that may be made by its manufacturer, is not guaranteed or endorsed by the publisher.
